# 7-*p*-Tolyl-10,11-dihydro­benzo[*h*]furo[3,4-*b*]quinolin-8(7*H*)-one

**DOI:** 10.1107/S160053681000591X

**Published:** 2010-02-20

**Authors:** Chunling Shi

**Affiliations:** a School of Chemistry and Chemical Engineering, Xuzhou Institute of Technology, Xuzhou 221008, People’s Republic of China

## Abstract

In the title compound, C_22_H_17_NO_2_, the fused ring system is essentially planar (r.m.s. deviation = 0.021 Å) and the dihedral angle between the dihydro­pyridine and tolyl rings is 80.98 (11)°. In the crystal, the mol­ecules are linked into chains along the *b* axis by inter­molecular N—H⋯O and C—H⋯O hydrogen bonds. Adjacent chains are linked by π–π inter­actions [centroid–centroid separation = 3.5748 (15) Å].

## Related literature

For the biological activity of podophyllotoxin and its derivatives, see: Bosmans *et al.* (1989 ▶); Eycken *et al.* (1989 ▶); Hitosuyanagi *et al.* (1997 ▶, 1999 ▶); Lienard *et al.* (1991 ▶); Magedov *et al.* (2007 ▶); Poli & Giambastiani (2002 ▶); Tomioka *et al.* (1989 ▶, 1993 ▶); Tratrat *et al.* (2002 ▶). For a related structure, see: Shi & Ji (2009 ▶).
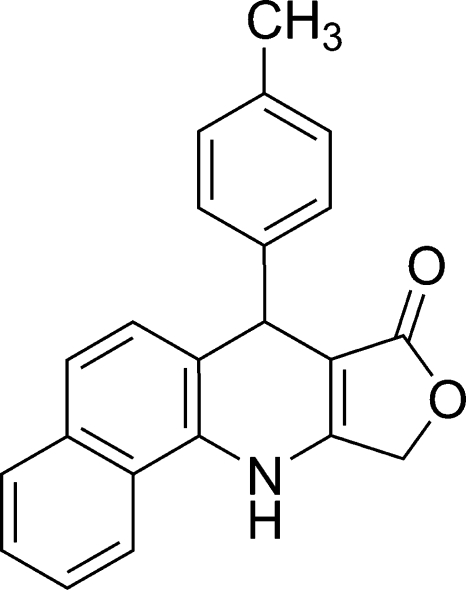


## Experimental

### 

#### Crystal data

C_22_H_17_NO_2_
*M* *_r_* = 327.37
Monoclinic, 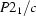

*a* = 10.6954 (16) Å
*b* = 13.0566 (18) Å
*c* = 12.183 (2) Å
β = 107.322 (3)°
*V* = 1624.1 (4) Å^3^
*Z* = 4
Mo *K*α radiation
μ = 0.09 mm^−1^
*T* = 223 K
0.60 × 0.34 × 0.30 mm

#### Data collection

Rigaku Mercury diffractometer
Absorption correction: multi-scan (Jacobson, 1998 ▶) *T*_min_ = 0.770, *T*_max_ = 0.975
15611 measured reflections
2978 independent reflections
2476 reflections with *I* > 2σ(*I*)
*R*_int_ = 0.043

#### Refinement

*R*[*F* ^2^ > 2σ(*F* ^2^)] = 0.064
*wR*(*F* ^2^) = 0.138
*S* = 1.18
2978 reflections
228 parameters
H-atom parameters constrained
Δρ_max_ = 0.22 e Å^−3^
Δρ_min_ = −0.18 e Å^−3^

### 

Data collection: *CrystalClear* (Rigaku, 2000 ▶); cell refinement: *CrystalClear*; data reduction: *CrystalStructure* (Rigaku/MSC, 2003 ▶); program(s) used to solve structure: *SHELXS97* (Sheldrick, 2008 ▶); program(s) used to refine structure: *SHELXL97* (Sheldrick, 2008 ▶); molecular graphics: *SHELXTL* (Sheldrick, 2008 ▶); software used to prepare material for publication: *SHELXTL*.

## Supplementary Material

Crystal structure: contains datablocks global, I. DOI: 10.1107/S160053681000591X/ci5030sup1.cif

Structure factors: contains datablocks I. DOI: 10.1107/S160053681000591X/ci5030Isup2.hkl

Additional supplementary materials:  crystallographic information; 3D view; checkCIF report

## Figures and Tables

**Table 1:** Hydrogen-bond geometry (Å, °)

*D*—H⋯*A*	*D*—H	H⋯*A*	*D*⋯*A*	*D*—H⋯*A*
N1—H1⋯O2^i^	0.87	2.00	2.802 (2)	153
C12—H12⋯O1^i^	0.94	2.49	3.248 (3)	137

Symmetry code: (i) 
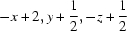
.

**Fig. 1.**
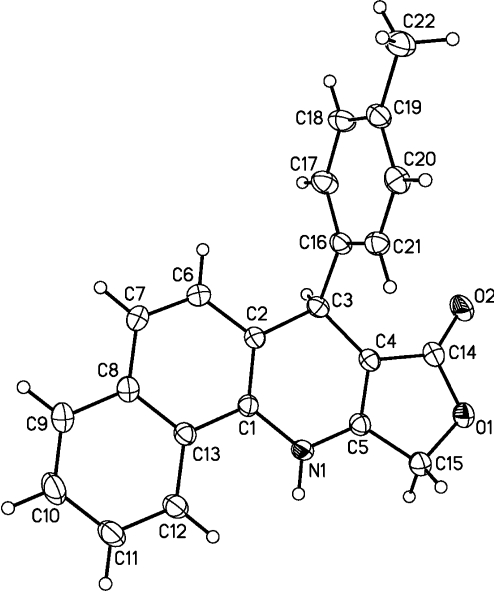
The molecular structure of the title compound, showing 30% probability displacement ellipsoids and the atom-numbering scheme.

**Fig. 2.**
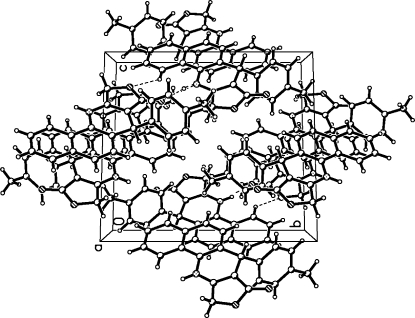
The crystal packing of the title compound.

## supplementary crystallographic information

### Comment

Podophyllotoxin is an antitumor lignan that inhibits microtubule assembly (Eycken *et al.*, 1989; Tomioka *et al.*, 1989; Bosmans *et al.*, 1989). Extensive structural modifications have been performed in order to obtain more potent and less toxic anticancer agents (Tomioka *et al.*, 1993; Lienard *et al.*, 1991; Poli *et al.*, 2002). Among them, 4-aza-podophllotoxin (9-aryl-4,9-dihydrofuro[3,4-b]quinolin-1(3*H*)-one) derivatives reported as powerful DNA topoisomerase II inhibitors, have recently attached considerable interest (Hitosuyanagi *et al.*, 1997; Hitosuyanagi *et al.*, 1999; Tratrat *et al.*,  2002; Magedov *et al.*, 2007). We report here the crystal structure of the title compound, which was synthesized by the three-component reaction of naphthalen-1-amine with 4-methylbenzaldehyde and tetronic acid catalyzed by L-proline using ethanol as solvent at 353 K.

In the title compound, the 1,4-dihydropyridine (C1–C5/N1) and furanone rings are planar (Fig. 1) and both are coplanar with the naphthalene ring system i.e the fused ring system is essentially planar (r.m.s. deviation 0.021 Å). The dihedral angle between C1–C5/N1 and C16–C21 planes is 80.98 (11)°. The conformation of the title molecule differs from that of a related molecule, 7-methyl-9-*p*-tolyl-4,9-dihydrofuro[3,4-b]quinolin-1(3*H*)-one (Shi *et al.*, 2009).

In the crystal structure, the molecules are linked by N1—H1···O2 and C12—H12···O1 intermolecular hydrogen bonds (Table 1) to form chains (Fig. 2) along the *b* axis. The adjacent chains are linked through π-π interactions between O1/C14/C4/C5/C15 and C1/C2/C6-C8/C13 rings with a centroid-centroid separation of 3.5748 (15) Å.

### Experimental

The title compound was prepared by the reaction of naphthalen-1-amine (1 mmol) and 4-methylbenzalhyde (1 mmol) with tetronic acid (1 mmol) in the presence of L-proline (0.1 mmol) in ethanol (2 ml) at 353 K. Crystals of the title compound suitable for X-ray diffraction were obtained by slow evaporation of a *N*,*N*-dimethylformamide and ethanol solution. ^1^H NMR (DMSO-d_6_, δ): 2.21 (3*H*, s, CH_3_), 4.98 (1*H*, d, J = 16.0 Hz, CH_2_), 5.07 (1*H*, d, J = 16.0 Hz, CH_2_), 5.13 (1*H*, s, CH), 7.05 (2*H*, d, J = 8.0 Hz, ArH), 7.12 (3*H*, dd, J_1_ = 6.4 Hz, J_2_ = 8.0 Hz, ArH), 7.46 (1*H*, d, J = 8.8 Hz, ArH), 7.51-7.55 (1*H*, m, ArH), 7.60-7.64 (1*H*, m, ArH), 7.84 (1*H*, d, J = 8.0 Hz, ArH), 8.21 (1*H*, d, J = 8.0 Hz, ArH), 10.22 (1*H*, s, NH).

### Refinement

H atoms were placed in calculated positions [N–H = 0.87 Å and C–H = 0.94-0.99 Å] and included in the final cycles of refinement using a riding model, with U_iso_(H) = 1.2-1.5 U_eq_(C).

### Crystal data

**Table:**

C_22_H_17_NO_2_	*F*(000) = 688
*M**_r_* = 327.37	*D*_x_ = 1.339 Mg m^−^^3^
Monoclinic, *P*2_1_/*c*	Mo *K*α radiation, λ = 0.71070 Å
Hall symbol: -P 2ybc	Cell parameters from 5302 reflections
*a* = 10.6954 (16) Å	θ = 3.0–25.3°
*b* = 13.0566 (18) Å	µ = 0.09 mm^−^^1^
*c* = 12.183 (2) Å	*T* = 223 K
β = 107.322 (3)°	Block, colourless
*V* = 1624.1 (4) Å^3^	0.60 × 0.34 × 0.30 mm
*Z* = 4	

### Data collection

**Table:**

Rigaku Mercury diffractometer	2978 independent reflections
Radiation source: fine-focus sealed tube	2476 reflections with *I* > 2σ(*I*)
graphite	*R*_int_ = 0.043
Detector resolution: 7.31 pixels mm^-1^	θ_max_ = 25.4°, θ_min_ = 3.1°
ω scans	*h* = −12→11
Absorption correction: multi-scan (Jacobson, 1998)	*k* = −15→12
*T*_min_ = 0.770, *T*_max_ = 0.975	*l* = −14→14
15611 measured reflections	

### Refinement

**Table:**

Refinement on *F*^2^	Primary atom site location: structure-invariant direct methods
Least-squares matrix: full	Secondary atom site location: difference Fourier map
*R*[*F*^2^ > 2σ(*F*^2^)] = 0.064	Hydrogen site location: inferred from neighbouring sites
*wR*(*F*^2^) = 0.138	H-atom parameters constrained
*S* = 1.18	*w* = 1/[σ^2^(*F*_o_^2^) + (0.0503*P*)^2^ + 0.6601*P*] where *P* = (*F*_o_^2^ + 2*F*_c_^2^)/3
2978 reflections	(Δ/σ)_max_ = 0.001
228 parameters	Δρ_max_ = 0.22 e Å^−^^3^
0 restraints	Δρ_min_ = −0.18 e Å^−^^3^

### Special details

**Table:**

Geometry. All esds (except the esd in the dihedral angle between two l.s. planes) are estimated using the full covariance matrix. The cell esds are taken into account individually in the estimation of esds in distances, angles and torsion angles; correlations between esds in cell parameters are only used when they are defined by crystal symmetry. An approximate (isotropic) treatment of cell esds is used for estimating esds involving l.s. planes.
Refinement. Refinement of *F*^2^ against ALL reflections. The weighted *R*-factor wR and goodness of fit *S* are based on *F*^2^, conventional *R*-factors *R* are based on *F*, with *F* set to zero for negative *F*^2^. The threshold expression of *F*^2^ > σ(*F*^2^) is used only for calculating *R*-factors(gt) etc. and is not relevant to the choice of reflections for refinement. *R*-factors based on *F*^2^ are statistically about twice as large as those based on *F*, and *R*- factors based on ALL data will be even larger.

### Fractional atomic coordinates and isotropic or equivalent isotropic displacement parameters (Å^2^)

**Table:**

	*x*	*y*	*z*	*U*_iso_*/*U*_eq_	
O1	1.04628 (16)	0.87892 (12)	0.19046 (14)	0.0446 (4)	
O2	1.02197 (17)	0.72658 (13)	0.26734 (16)	0.0520 (5)	
N1	0.88595 (17)	1.05854 (13)	0.32900 (16)	0.0357 (5)	
H1	0.8921	1.1200	0.3035	0.043*	
C1	0.8218 (2)	1.04144 (15)	0.41266 (18)	0.0303 (5)	
C2	0.8127 (2)	0.94420 (16)	0.45353 (18)	0.0321 (5)	
C3	0.8734 (2)	0.84879 (15)	0.41573 (19)	0.0323 (5)	
H3	0.9455	0.8252	0.4827	0.039*	
C4	0.9332 (2)	0.88088 (16)	0.32405 (18)	0.0327 (5)	
C5	0.9376 (2)	0.97791 (16)	0.28925 (19)	0.0330 (5)	
C6	0.7450 (2)	0.93198 (18)	0.5358 (2)	0.0442 (6)	
H6	0.7384	0.8661	0.5648	0.053*	
C7	0.6888 (3)	1.01196 (18)	0.5748 (2)	0.0481 (7)	
H7	0.6436	1.0004	0.6291	0.058*	
C8	0.6977 (2)	1.11247 (17)	0.5345 (2)	0.0398 (6)	
C9	0.6407 (3)	1.19767 (19)	0.5729 (2)	0.0518 (7)	
H9	0.5941	1.1878	0.6264	0.062*	
C10	0.6521 (3)	1.2934 (2)	0.5338 (3)	0.0570 (7)	
H10	0.6140	1.3493	0.5605	0.068*	
C11	0.7207 (3)	1.30888 (18)	0.4538 (2)	0.0515 (7)	
H11	0.7296	1.3755	0.4279	0.062*	
C12	0.7747 (2)	1.22844 (16)	0.4130 (2)	0.0413 (6)	
H12	0.8189	1.2400	0.3580	0.050*	
C13	0.7652 (2)	1.12779 (16)	0.45230 (19)	0.0330 (5)	
C14	1.0009 (2)	0.81812 (18)	0.2636 (2)	0.0392 (6)	
C15	1.0079 (2)	0.98353 (17)	0.2007 (2)	0.0410 (6)	
H15A	0.9500	1.0088	0.1274	0.049*	
H15B	1.0846	1.0284	0.2258	0.049*	
C16	0.7751 (2)	0.76137 (15)	0.38090 (19)	0.0332 (5)	
C17	0.7769 (2)	0.68286 (17)	0.4572 (2)	0.0436 (6)	
H17	0.8420	0.6822	0.5288	0.052*	
C18	0.6844 (3)	0.60477 (17)	0.4302 (2)	0.0463 (6)	
H18	0.6875	0.5527	0.4842	0.056*	
C19	0.5881 (2)	0.60229 (17)	0.3254 (2)	0.0395 (6)	
C20	0.5876 (2)	0.68095 (17)	0.2488 (2)	0.0407 (6)	
H20	0.5239	0.6809	0.1765	0.049*	
C21	0.6785 (2)	0.75938 (17)	0.2760 (2)	0.0376 (5)	
H21	0.6747	0.8121	0.2225	0.045*	
C22	0.4884 (3)	0.51737 (19)	0.2961 (3)	0.0541 (7)	
H22A	0.5015	0.4722	0.3618	0.081*	
H22B	0.4985	0.4788	0.2312	0.081*	
H22C	0.4010	0.5464	0.2763	0.081*	

### Atomic displacement parameters (Å^2^)

**Table:**

	*U*^11^	*U*^22^	*U*^33^	*U*^12^	*U*^13^	*U*^23^
O1	0.0519 (10)	0.0402 (9)	0.0490 (10)	0.0012 (7)	0.0264 (9)	−0.0075 (8)
O2	0.0626 (12)	0.0345 (10)	0.0664 (12)	0.0061 (8)	0.0305 (10)	−0.0097 (8)
N1	0.0415 (11)	0.0252 (9)	0.0463 (12)	0.0009 (8)	0.0222 (9)	0.0003 (8)
C1	0.0299 (11)	0.0312 (12)	0.0313 (12)	−0.0018 (9)	0.0113 (9)	−0.0033 (9)
C2	0.0358 (12)	0.0297 (12)	0.0310 (12)	−0.0014 (9)	0.0101 (10)	−0.0026 (9)
C3	0.0336 (12)	0.0266 (11)	0.0348 (12)	0.0022 (8)	0.0076 (10)	0.0009 (9)
C4	0.0306 (12)	0.0307 (12)	0.0357 (13)	−0.0018 (9)	0.0084 (10)	−0.0052 (9)
C5	0.0316 (12)	0.0320 (12)	0.0367 (13)	−0.0003 (9)	0.0119 (10)	−0.0032 (9)
C6	0.0605 (16)	0.0341 (13)	0.0444 (15)	−0.0012 (11)	0.0254 (12)	0.0034 (11)
C7	0.0632 (17)	0.0431 (14)	0.0503 (16)	0.0005 (12)	0.0355 (14)	−0.0009 (11)
C8	0.0432 (14)	0.0387 (13)	0.0404 (13)	0.0012 (10)	0.0171 (11)	−0.0048 (10)
C9	0.0601 (17)	0.0476 (16)	0.0573 (17)	0.0051 (12)	0.0321 (14)	−0.0091 (12)
C10	0.0614 (18)	0.0427 (16)	0.075 (2)	0.0090 (12)	0.0321 (16)	−0.0141 (13)
C11	0.0565 (16)	0.0305 (13)	0.0739 (19)	0.0039 (11)	0.0291 (14)	−0.0036 (12)
C12	0.0426 (13)	0.0309 (12)	0.0540 (16)	0.0001 (10)	0.0198 (12)	−0.0020 (11)
C13	0.0313 (12)	0.0312 (12)	0.0368 (13)	−0.0018 (9)	0.0108 (10)	−0.0039 (9)
C14	0.0393 (13)	0.0370 (14)	0.0409 (14)	−0.0001 (10)	0.0114 (11)	−0.0074 (10)
C15	0.0456 (14)	0.0368 (13)	0.0450 (15)	−0.0006 (10)	0.0201 (11)	−0.0048 (10)
C16	0.0366 (12)	0.0256 (11)	0.0379 (13)	0.0019 (9)	0.0118 (10)	−0.0013 (9)
C17	0.0518 (15)	0.0333 (13)	0.0394 (14)	−0.0025 (11)	0.0038 (11)	0.0035 (10)
C18	0.0605 (16)	0.0293 (12)	0.0495 (16)	−0.0050 (11)	0.0168 (13)	0.0046 (10)
C19	0.0372 (13)	0.0318 (12)	0.0523 (15)	−0.0022 (9)	0.0178 (11)	−0.0070 (10)
C20	0.0358 (13)	0.0414 (13)	0.0410 (14)	−0.0001 (10)	0.0056 (10)	−0.0050 (11)
C21	0.0418 (13)	0.0320 (12)	0.0383 (14)	0.0018 (10)	0.0109 (11)	0.0027 (10)
C22	0.0510 (15)	0.0437 (15)	0.071 (2)	−0.0125 (12)	0.0231 (14)	−0.0085 (13)

### Geometric parameters (Å, °)

**Table:**

O1—C14	1.385 (3)	C9—H9	0.94
O1—C15	1.442 (3)	C10—C11	1.398 (4)
O2—C14	1.215 (3)	C10—H10	0.94
N1—C5	1.344 (3)	C11—C12	1.362 (3)
N1—C1	1.406 (3)	C11—H11	0.94
N1—H1	0.87	C12—C13	1.413 (3)
C1—C2	1.378 (3)	C12—H12	0.94
C1—C13	1.430 (3)	C15—H15A	0.98
C2—C6	1.409 (3)	C15—H15B	0.98
C2—C3	1.538 (3)	C16—C17	1.380 (3)
C3—C4	1.503 (3)	C16—C21	1.384 (3)
C3—C16	1.524 (3)	C17—C18	1.390 (3)
C3—H3	0.99	C17—H17	0.94
C4—C5	1.341 (3)	C18—C19	1.382 (3)
C4—C14	1.433 (3)	C18—H18	0.94
C5—C15	1.489 (3)	C19—C20	1.387 (3)
C6—C7	1.359 (3)	C19—C22	1.506 (3)
C6—H6	0.94	C20—C21	1.383 (3)
C7—C8	1.414 (3)	C20—H20	0.94
C7—H7	0.94	C21—H21	0.94
C8—C13	1.413 (3)	C22—H22A	0.97
C8—C9	1.413 (3)	C22—H22B	0.97
C9—C10	1.356 (4)	C22—H22C	0.97
			
C14—O1—C15	108.88 (16)	C11—C12—C13	120.8 (2)
C5—N1—C1	118.59 (18)	C11—C12—H12	119.6
C5—N1—H1	120.7	C13—C12—H12	119.6
C1—N1—H1	120.7	C8—C13—C12	118.4 (2)
C2—C1—N1	120.70 (18)	C8—C13—C1	119.01 (19)
C2—C1—C13	121.46 (19)	C12—C13—C1	122.6 (2)
N1—C1—C13	117.83 (18)	O2—C14—O1	119.4 (2)
C1—C2—C6	117.90 (19)	O2—C14—C4	131.3 (2)
C1—C2—C3	123.82 (18)	O1—C14—C4	109.28 (19)
C6—C2—C3	118.28 (19)	O1—C15—C5	103.55 (18)
C4—C3—C16	114.39 (18)	O1—C15—H15A	111.1
C4—C3—C2	107.95 (17)	C5—C15—H15A	111.1
C16—C3—C2	111.84 (17)	O1—C15—H15B	111.1
C4—C3—H3	107.5	C5—C15—H15B	111.1
C16—C3—H3	107.5	H15A—C15—H15B	109.0
C2—C3—H3	107.5	C17—C16—C21	117.7 (2)
C5—C4—C14	107.9 (2)	C17—C16—C3	119.9 (2)
C5—C4—C3	124.11 (19)	C21—C16—C3	122.32 (19)
C14—C4—C3	127.9 (2)	C16—C17—C18	121.3 (2)
C4—C5—N1	124.7 (2)	C16—C17—H17	119.4
C4—C5—C15	110.36 (19)	C18—C17—H17	119.4
N1—C5—C15	124.93 (19)	C19—C18—C17	121.2 (2)
C7—C6—C2	122.4 (2)	C19—C18—H18	119.4
C7—C6—H6	118.8	C17—C18—H18	119.4
C2—C6—H6	118.8	C18—C19—C20	117.2 (2)
C6—C7—C8	120.6 (2)	C18—C19—C22	121.2 (2)
C6—C7—H7	119.7	C20—C19—C22	121.7 (2)
C8—C7—H7	119.7	C21—C20—C19	121.7 (2)
C13—C8—C9	119.0 (2)	C21—C20—H20	119.1
C13—C8—C7	118.6 (2)	C19—C20—H20	119.1
C9—C8—C7	122.4 (2)	C20—C21—C16	120.9 (2)
C10—C9—C8	121.1 (2)	C20—C21—H21	119.6
C10—C9—H9	119.4	C16—C21—H21	119.6
C8—C9—H9	119.4	C19—C22—H22A	109.5
C9—C10—C11	119.9 (2)	C19—C22—H22B	109.5
C9—C10—H10	120.0	H22A—C22—H22B	109.5
C11—C10—H10	120.0	C19—C22—H22C	109.5
C12—C11—C10	120.7 (2)	H22A—C22—H22C	109.5
C12—C11—H11	119.6	H22B—C22—H22C	109.5
C10—C11—H11	119.6		
			
C5—N1—C1—C2	0.4 (3)	C9—C8—C13—C1	−179.4 (2)
C5—N1—C1—C13	−178.79 (19)	C7—C8—C13—C1	0.1 (3)
N1—C1—C2—C6	−178.9 (2)	C11—C12—C13—C8	0.3 (4)
C13—C1—C2—C6	0.3 (3)	C11—C12—C13—C1	−179.3 (2)
N1—C1—C2—C3	1.9 (3)	C2—C1—C13—C8	−0.5 (3)
C13—C1—C2—C3	−178.92 (19)	N1—C1—C13—C8	178.7 (2)
C1—C2—C3—C4	−3.7 (3)	C2—C1—C13—C12	179.1 (2)
C6—C2—C3—C4	177.1 (2)	N1—C1—C13—C12	−1.7 (3)
C1—C2—C3—C16	−130.4 (2)	C15—O1—C14—O2	179.3 (2)
C6—C2—C3—C16	50.4 (3)	C15—O1—C14—C4	−0.6 (2)
C16—C3—C4—C5	128.9 (2)	C5—C4—C14—O2	179.8 (3)
C2—C3—C4—C5	3.7 (3)	C3—C4—C14—O2	3.2 (4)
C16—C3—C4—C14	−54.9 (3)	C5—C4—C14—O1	−0.3 (3)
C2—C3—C4—C14	179.9 (2)	C3—C4—C14—O1	−176.95 (19)
C14—C4—C5—N1	−178.8 (2)	C14—O1—C15—C5	1.1 (2)
C3—C4—C5—N1	−2.0 (4)	C4—C5—C15—O1	−1.3 (3)
C14—C4—C5—C15	1.0 (3)	N1—C5—C15—O1	178.54 (19)
C3—C4—C5—C15	177.8 (2)	C4—C3—C16—C17	137.1 (2)
C1—N1—C5—C4	−0.4 (3)	C2—C3—C16—C17	−99.8 (2)
C1—N1—C5—C15	179.8 (2)	C4—C3—C16—C21	−45.9 (3)
C1—C2—C6—C7	0.4 (4)	C2—C3—C16—C21	77.3 (3)
C3—C2—C6—C7	179.6 (2)	C21—C16—C17—C18	−0.5 (3)
C2—C6—C7—C8	−0.8 (4)	C3—C16—C17—C18	176.7 (2)
C6—C7—C8—C13	0.5 (4)	C16—C17—C18—C19	0.7 (4)
C6—C7—C8—C9	−180.0 (2)	C17—C18—C19—C20	−0.1 (4)
C13—C8—C9—C10	−1.3 (4)	C17—C18—C19—C22	179.7 (2)
C7—C8—C9—C10	179.2 (3)	C18—C19—C20—C21	−0.7 (3)
C8—C9—C10—C11	0.3 (4)	C22—C19—C20—C21	179.5 (2)
C9—C10—C11—C12	1.0 (4)	C19—C20—C21—C16	0.9 (3)
C10—C11—C12—C13	−1.3 (4)	C17—C16—C21—C20	−0.3 (3)
C9—C8—C13—C12	1.0 (3)	C3—C16—C21—C20	−177.4 (2)
C7—C8—C13—C12	−179.5 (2)		

### Hydrogen-bond geometry (Å, °)

**Table:**

*D*—H···*A*	*D*—H	H···*A*	*D*···*A*	*D*—H···*A*
N1—H1···O2^i^	0.87	2.00	2.802 (2)	153
C12—H12···O1^i^	0.94	2.49	3.248 (3)	137

Symmetry codes: (i) −*x*+2, *y*+1/2, −*z*+1/2.

## References

[bb1] Bosmans, J. P., Eycken, J. V. & Vandewalle, M. (1989). *Tetrahedron Lett.* **30**, 3877–3880.

[bb2] Eycken, J. V., Bosmans, J. P., Haver, D. V. & Vandewalle, M. (1989). *Tetrahedron Lett.* **30**, 3873–3876.

[bb3] Hitosuyanagi, Y., Kobayashi, M., Fukuyo, M., Takeya, K. & Itokawa, H. (1997). *Tetrahedron Lett.* **38**, 8295–8296.

[bb4] Hitosuyanagi, Y., Kobayashi, M., Morita, H., Itokawa, H. & Takeya, K. (1999). *Tetrahedron Lett.* **40**, 9107–9110.

[bb5] Jacobson, R. (1998). Private communication to the Rigaku Corporation, Tokyo, Japan.

[bb6] Lienard, P., Royer, J., Quirion, J. C. & Husson, H. P. (1991). *Tetrahedron Lett.* **32**, 2489–2492.

[bb7] Magedov, I. V., Manapadi, M., Rozhkova, E., Przheval’skii, N. M., Rogelj, S., Shors, S. T., Steelant, W. A., Slambrouck, S. V. & Korinienko, A. (2007). *Bioorg. Med. Chem. Lett.* **17**, 1381–1385. 10.1016/j.bmcl.2006.11.095 PMC3393043 17188868

[bb8] Poli, G. & Giambastiani, G. (2002). *J. Org. Chem.* **67**, 9456–9459. 10.1021/jo026068+ 12492354

[bb9] Rigaku (2000). *CrystalClear.* Rigaku Corporation, Tokyo, Japan.

[bb10] Rigaku/MSC (2003). *CrystalStructure.* Rigaku/MSC, The Woodlands, Texas, USA.

[bb11] Sheldrick, G. M. (2008). *Acta Cryst.* A**64**, 112–122. 10.1107/S0108767307043930 18156677

[bb12] Shi, C. & Ji, M. (2009). *Acta Cryst.* E**65**, o100. 10.1107/S1600536808041457 PMC2968025 21581564

[bb13] Tomioka, K., Kubota, Y. & Koga, K. (1989). *Tetrahedron Lett.* **30**, 2953–2954.

[bb14] Tomioka, K., Kubota, Y. & Koga, K. (1993). *Tetrahedron*, **49**, 1891–1900.

[bb15] Tratrat, C., Renault, S. G. & Husson, H. P. (2002). *Org. Lett.* **4**, 3187–3189. 10.1021/ol0200908 12227745

